# A Novel Coordinated Edge Caching with Request Filtration in Radio Access Network

**DOI:** 10.1155/2013/654536

**Published:** 2013-12-29

**Authors:** Yang Li, Yuemei Xu, Tao Lin, Xiaohui Wang, Song Ci

**Affiliations:** ^1^High Performance Network Laboratory, Institute of Acoustics, Chinese Academy of Sciences, Beijing 100190, China; ^2^School of Computer Science and Technology, Nanjing Normal University, Nanjing 210023, China; ^3^Department of Computer and Electronics Engineering, University of Nebraska-Lincoln, Omaha, NE 68182, USA

## Abstract

Content caching at the base station of the Radio Access Network (RAN) is a way to reduce backhaul transmission and improve the quality of experience. So it is crucial to manage such massive microcaches to store the contents in a coordinated manner, in order to increase the overall mobile network capacity to support more number of requests. We achieve this goal in this paper with a novel caching scheme, which reduces the repeating traffic by request filtration and asynchronous multicast in a RAN. Request filtration can make the best use of the limited bandwidth and in turn ensure the good performance of the coordinated caching. Moreover, the storage at the mobile devices is also considered to be used to further reduce the backhaul traffic and improve the users' experience. In addition, we drive the optimal cache division in this paper with the aim of reducing the average latency user perceived. The simulation results show that the proposed scheme outperforms existing algorithms.

## 1. Introduction

With the worldwide growth in the adoption of smart phones and tablets, access to Internet from mobile devices (MDs) is projected to grow very significantly [1]. When Internet is accessed by an MD, the content has to be fetched from the original servers outside of the mobile networks. The CDNs [2, 3] help reduce Internet bandwidth consumption and associated delay/jitter, but the content must additionally travel through the wireless carrier Core Network (CN) and Radio Access Network (RAN) before reaching the MD. Bringing each of the requested contents from the CDNs can put significant strain on the carrier's CN and RAN backhaul, leading to congestion, significant delay, and constraint on the network's capacity to serve large number of concurrent content requests.

Concurrently, the emergence of massive content delivery involves repeated wireless transmission of contents that are required multiple times by different users in a completely asynchronous way. Since the relative delay at which two users may require the same content is generally much larger than the duration of the file download, a conventional network architecture treats each session as independent data, and it is therefore incapable of exploiting the intrinsic multicasting capability of the wireless medium. Traditional methods for increasing data throughput in wireless networks have relied on the following three approaches [4]: (i) increase of spectrum usage, (ii) increase of the per-link spectral efficiency, and (iii) increase of spatial reuse. Increasing the amount of spectrum is limited by the fact that spectrum is a finite resource, and allocating new bands to cellular services is a long and expensive process. Increasing the spectral efficiency per link is also approaching its limits: fourth generation cellular systems such as LTE have a near-optimal physical layer, using OFDM together with capacity approaching codes and multiple antenna elements. While further improvements, such as cooperative multipoint and interference alignment, are expensive and/or require redesign of the physical layer. In addition, all three approaches require every base station (BS) to have a high-speed backhaul, whose quality must be better than the aggregate data rate of all its served users. It is not practical since the infrastructure promotion is bound to lag behind Internet traffic growth.

To facilitate the tremendous growth of mobile Internet traffic consumption without the above associated limitations, in this paper we are describing a radically new approach that is based on the following two key observations: (i) a large amount of content delivery traffic is caused by a few, popular files and (ii) disk storage is a quantity that increases faster than any other component in communications or processing systems. The capabilities of storage devices have been further enhanced by improved coding for storage, in particular excellent distributed storage codes. The essential idea of our approach thus is to trade off backhaul capacity with caching of contents at local BSs at the edge of the RAN. In other words, those BSs obtain the most popular video files by downloading through their weak backhaul links, such that most requests can be served from the RAN caches, instead of having to be fetched from the Internet CDNs.

**Figure 1:**
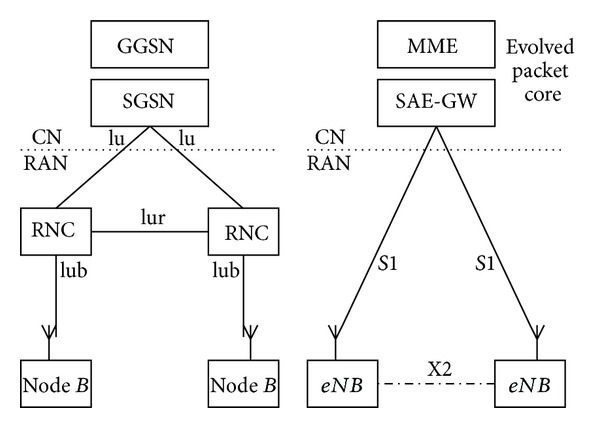
The RAN evolution from 3G to LTE.

Now the mobile network is undergoing an evolution from 3G to LTE, as shown in Figure 1 [5]. In such flattened network architecture, there are more eNodeBs than before under a centralized SAE-GW control directly. Since the size of edge caches in RAN is much smaller than that of caches used in Internet CDN, it is preferable to store contents at these massive BSs in a coordinated manner, which allows more diverse contents to be efficiently cached in a RAN. The controller SAE-GW, therefore, can leverage these storage resources to serve domestic request in a managed peer-to-peer fashion, thus improving the overall content delivery performance. Ideally, one would like to *optimize the design of this coordinated caching in order to maximize the amount of the supported request in the RAN*. There are many coordinated caching algorithms proposed based on optimization methods, where each content request arriving rate is required to be known in advance [6–8]. However, in practice, *the content request rate demanded by users is varying over time with its popularity change*, making the optimal scheme derived from the past statistic period not applicable anymore. Furthermore, it is complex and not cost effective to collect all content request rates at SAE-GW.

In addition, *due to the independent peer-to-peer unicast transmission mechanism, massive repeating traffics flood the network, consuming the precious link bandwidth resource.* For example, two MDs may fetch the same content from the same neighbor BS concurrently. In contrast, *merging these two traffic flows into one can greatly reduce the total network traffic delivered.* Therefore, we propose a novel caching strategy, based on new concepts introduced in the paper: *request filtration* and *asynchronous multicast*. As a result, one copy of the cached content is able to serve all users within one RAN. The bandwidth requirement, thereby, is derived from the shared cache size accordingly.

Our concept is pushed even further by introducing the notion of MDs themselves as cache nodes. Recent years have seen an enormous proliferation of smartphones and tablets that have anywhere between 10 and 64 GByte of storage (even 500 GByte on typical laptop hard disks). *By enabling communications among devices through base station in a p2p manner, the ensemble of MDs can become a distributed cache that allows much more efficient downloading.* The advantage of using MDs lies in the small deployment costs and automatic upscaling of the capacity as the density of such devices increases. The drawback lies in the necessity to motivate users to participate in the cache and the more random nature of the available throughput. For MDs as caches, some of the fundamental questions are (i) what files should be stored by the MDs? (ii) How can they most efficiently acquire those files? And (iii) does a centralized control of the communications through base station have significant advantages?

We summarize the main contributions of this paper as follows.

- We divide the original cooperation problem into subproblems that focus on the direct or indirect cache connection according to the practical topology.
- Based on a deep analysis on how the request filtration can reduce the total network traffic, we investigate the relationship between the shared cache size and the link bandwidth.
- Combined with the caches provided by the MDs and the caches at the base station, we propose our complete coordinated edge caching strategy.
- With the aim of reducing the average latency user perceived, we derive the optimal shared cache size for the proposed coordinated edge caching strategy, without knowing the content request rate in advance.
- We evaluate the effectiveness of the proposed scheme by extensive simulation experiments. The results show that our scheme outperforms existing schemes.

The remainder of this paper is organized as follows. Sections 2 and 3 present the related works and motivation, respectively. Based on the request filtration analysis, Section 4 illustrates the relationship between the shared cache size and link bandwidth; then proposes the indirect coordinated edge caching strategy Section 5.  Section 6 derives the optimal shared cache size under the proposed strategy. Scheme evaluation results through simulation are presented in Section 7. Finally, Section 8 offers some concluding remarks.

## 2. Related Work

Content caching has been a key component of Internet based services for many years [3], and there have been many studies in the literature on content caching techniques [9–11]. In particular, coordinated content caching has been studied extensively [12–14]. Researchers have investigated the effectiveness of collaborative caching and proposed numerous collaborative caching schemes [15–17]. Additionally, there has been some research in content caching in wireless networks [18, 19], such as hoc networks [20–22], mobile broadcast environment [23], and MDs [4, 24–26]. Especially with the Information Centric Networking (ICN) resurgence in recent years [27, 28], the in-network caching attracts more research attention [29–33].

A promising evolution of the content delivery architecture consists of extending the caches to the “last mile” by incorporating small servers close to the edge of the network. For instance, this approach leverages devices at the AP and BS, or within users' homes, such as advocated by the Nanodatacenters [34] consortium, network attached storage (NAS) such as Boxee [35], or appliances promoted by business initiatives such as Apple TV. A scalable and adaptive mechanism designed for such “diffuse cloud” of nanoservers is proposed in [36], where part of or the entire storage and bandwidth capacities of set-top devices can be leased to a CDN or a content provider. The latter leverage these resources to store content and serve download requests effectively in a managed peer-to-peer fashion. Moreover, a new system for a neighborhood-assisted video-on-demand service, in [37], is designed to reduce access link traffic by carefully placing VoD data across the neighborhood. So the access network load can be relieved by local connectivity and storage in residential environments. Especially in [38], Fayazbakhsh et al. argue that the simple edge-based caching architecture can achieve a competitive good performance, compared to a full-fledged ICN architecture (i.e., with pervasive caches and nearest-replica routing) with respect to response time, network congestion, and origin server load. Using sensitivity analysis on a range of configuration parameters, they find that the optimistic best-case improvement that ICN can provide is 17% over the simple edge caching architecture (on all metrics).

In addition, AT&T [19] provides a simple cost model for cellular networks, which the third parties can easily use to determine the cost-benefit tradeoffs for their own cellular network settings. It is a first large scale caching analysis for cellular networks, which explored the potential of forward caching in 3G cellular networks by using traffic traces generated by millions of users from one of the world's largest 3G cellular networks. This cost model shows the tradeoffs between deploying forward caching at different levels in the 3G network hierarchy. They also draw a conclusion that caching at regional data centers is the most beneficial with a 26.7% savings in cost.

Our work differs from the above studies in three ways. First, we address the request filtration in order to reduce the total network traffic. To the best of our knowledge, our work is the first attempt to formally investigate and provide insights in addressing these issues. Secondly, we derive a model to define the relationship between the shared cache size and the upstream bandwidth requirement. Thirdly, we investigate the cache space provided by the users in our caching coordinated strategy in order to maximize the local supported traffic.

## 3. Coordinated Edge Caching Mechanism

Based on the topology derived from the LTE system, as shown in Figure 1, we divide the original problem into subproblems that focus on the cooperation in different cache levels. We first discuss the coordination among interconnected caches at the edge BSs. This is derived from the practical scenario where neighbor edge BSs are directly connected by high capacity links (known as X2 traffic). The coordination among edge BSs connected via the SAE-GW will then be investigated. Such a form of cooperation is achieved through the unique downstream content retrieval with dynamic routing decisions.

### 3.1. Direct Edge Caches Coordination

**Figure 2:**
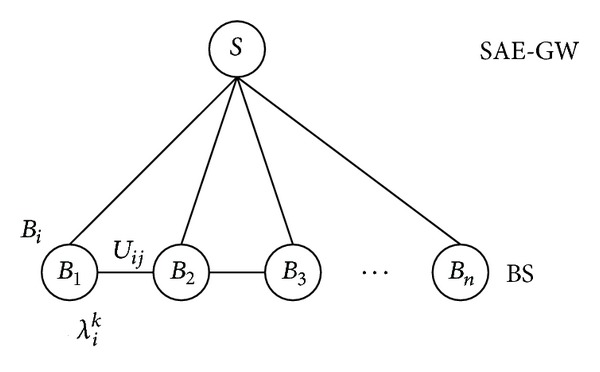
Direct coordinated edge caching model.

As denoted by Figure 2, fully interconnected caches at the edge BSs are able to disseminate contents via direct links. We then investigate the subproblem that aims to maximize the amount of supported traffic at this level by utilizing the capacity of direct links.

We use *S* and *B*_*i*_ to present SAE-GW and BS, respectively, where each *B*_*i*_,  *i* ∈ *n* has storage capacity *c*_*i*_. The link capacity between a pair of BSs at the edge caches is presented by *U*_*ij*_. We define the average arrival rate of content requests at edge cache *C*_*i*_ as *λ*_*i*_. The proportion of requests that attempt to access content *k* ∈ *K* is represented by *p*_*k*_, and we assume that such a distribution pattern is uniform for all fully connected caches colocated in the same domain. Thus the rate of a request to content *k* at cache is represented by *λ*_*i*_^*k*^ = *λ*_*i*_ *p*_*k*_. Combined with concerns about the segment size, we treat *λ*_*i*_^*k*^ *s*_*k*_ as the bandwidth requirement of requests to content *k* in one time unit. Without loss of generality, we assume that the link capacity *U*_*ij*_ is consistently larger than ∑_*k*∈*K*_ *λ*_*i*_^*k*^ *s*_*k*_,  *i* ∈ *n*. The assumption is practical based on our observations in the real world system. This leads to the following theorem, which is used in cache cooperation of interconnected edge caches.

**Theorem 1**

Given ∑_*i*∈*n*_ *c*_*i*_ ≤ ∑_*k*∈*K*_ *s*_*k*_ and *U*_*ij*_ ≥ ∑_*k*∈*K*_ *λ*_*i*_^*k*^ *s*_*k*_, for any content *k*, there is at most one copy of the content *k* in the total *n* caches, in order to maximize the satisfied requests.

**Proof**

Suppose there are two copies of a content *k* in caches at edges *B*_*i*_ and *B*_*j*_, *i* ≠ *j*. We delete one copy stored in the cache at edge *B*_*j*_, which generates an assignment not worse than the optimal solution. Given ∑_*i*∈*n*_ *c*_*i*_ ≤ ∑_*k*∈*K*_ *s*_*k*_, we can store another content *k*′ in the empty cache room at edge *B*_*j*_. Since *U*_*ij*_ ≥ ∑_*k*∈*K*_ *λ*_*i*_^*k*^ *s*_*k*_, the new cached content *k*′ can provide service in the domain, which yields an assignment that surpasses the optimal solution. This is a contradiction.

Theorem 1 suggests that all duplicated contents in the interconnected caches set need to be removed and replaced by other unavailable contents. Moreover, all directly connected caches can be treated as one single cache. There is no need to consider the strategic placement of contents with such a combination, since the traffic can be easily routed through direct links at the edge BSs.

### 3.2. Motivation for Indirect Edge Caches Coordination

If the direct link between the edge BSs is not available or the direct link is not used for data (only for signaling), the coordination among these caches can be realized with the aid of SAE-GW. If the content is available at the accessed BS, the request could be immediately served by the attached cache. When the request is not fulfilled, it will then be sent to the corresponding upper level SAE-GW. Whether the request is routed to the other bottom caches or the original server depends on the cached content category and the reverse link capacity from BS to SAE-GW. The efficiency of the indirect edge caches coordination is complicate, so we first give an *illustrate example* in order to make the problem easily understood. Then our proposed indirect cache coordination strategy is presented in the next following two sections.

**Figure 3:**
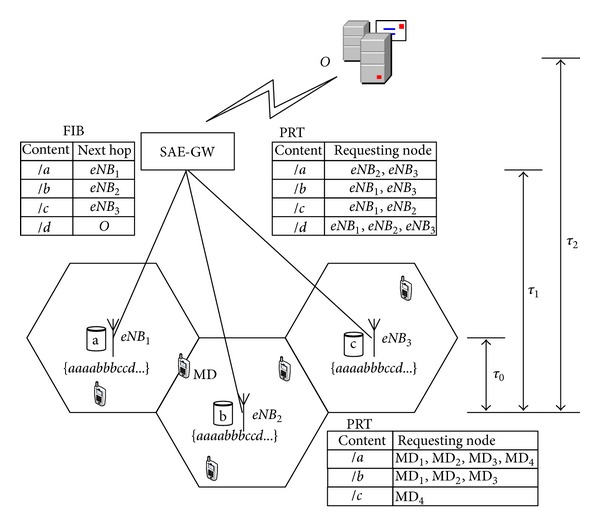
A motivating example.

Figure 3 shows a RAN consisting of three BSs, *eNB*_1_, *eNB*_2_, and *eNB*_3_, and one original server *O* serving four content objects, *a*, *b*, *c*, and *d*. Moreover, all BSs have storage capacity to store one single content object only, whereas SAE-GW does not have any available capacity for storing contents. In addition, the capacity of each upstream link from the BS to the SAE-GW is only capable of fetching one single content object simultaneously.

We assume that there are sets of MDs sending request flows to their BSs at each cell. The Request flows are identical, represented by a repeating sequence {*aaaabbbccd*}. We assume that the performance (e.g., latency) of fetching contents from a peer BS in the same RAN is much better than from the original server *O*, since in real network, the origin may reside quite far away from the network. Then, apparently, storing distinct contents at the BSs would reduce the overall delay and improve the network performance that MDs experience. However, the performance improvement is subject to the BS's upstream link capacity when we consider the bandwidth consumed during the delivery of the massive contents. Due to the limited storage and link capacities, the problem is *how to select contents to store at each router so as to improve the network performance*.

We consider the following three edge caching strategies. (1)   *Noncoordinated caching*: all BSs work independently, where they both adopt the canonical caching policy based on frequency or historical usage. Assume that the content popularity distribution is consistent, and all BSs have already cumulated the information that content *a* is requested more often than *b*, *c*, and *d*. In this case, all BSs store *a*. (2) *Coordinated caching*: all BSs work jointly and always prefer each other over the original server whenever possible. In this case, each BS may store different content, respectively. Without loss of generality, we assume that *eNB*_1_ stores *a*, *eNB*_2_ stores *b*, and *eNB*_3_ stores *c*. Then, on cache misses, a requested content will always be retrieved from peer BSs and then the original server *O*, sequentially. (3)  *Request filtration*: the content caching and searching are the same as in the coordinated caching strategy. However, at each node (BS and SAE-GW) in a RAN, all unsatisfied requests for the same content are filtered into one request only (i.e., except the first request being sent out, all the following unsatisfied requests for the same content, before the reply returns, are filtered by the pending request table (PRT)). Through adding the requesting nodes to the corresponding content entry in the PRT built by its ancestor, the returned content is multicast to all record requesters, as shown in Figure 3.

We compare the above three edge caching strategies by using two metrics: the *load on origin* and the *traffic delivered*. We summarize the comparison results in Table 1.

First of all, the load on origin is measured by the percentage of all requests served directly by the original server *O*. With the noncoordinated strategy, the requests for content *a* will be directly served by the accessed BSs (recall that all BSs store *a* in this case), while the requests for *b*, *c*, and *d* will have to be served by the original server. This means a total 6/10 of all Requests from three flows incur the traffic load on the original server. However, with the coordinated and request filtration strategies, since *a*, *b*, and *c* are stored locally, requests from the MDs can be served by all BSs.

The performance difference between coordinated caching and request filtration is determined by the average latency of fetching contents and the request arriving rate. As shown in Figure 3, we denote by *τ*_1_ the average latency of serving requests from a peer BS in the given RAN and by *λ*_*i*_^*k*^ the average request arriving rate for content *k* in one cell. The case of *λ*_*i*_^*k*^ · *τ*_1_ ≥ 1 means that there are some requests for content *k* arrived at BS before the previous Reply for the same content returns back. In this case, with the request filtration strategy, the BS filters the following requests and records the requesting nodes to the PRT (the specific operation is explained in Section 3.1), while with the coordinated strategy, all requests are sent out either to the peer BS or to the original server if the upstream link of the peer BS is busy.

Therefore, with the coordinated strategy, in the case of requests for the *a*, *b*, and *c* being all served in the RAN (*best case*) due to their lower request rate, the total 3/30 for *d* of all requests from three flows incurs the traffic load on the original server. However, in the case of all the upstream link being busy if all requests arrive simultaneously (*worst case*), the link capacity can serve one request only, so other requests for *a*, *b*, and *c* are still delivered to the original server. Hence, the load on the origin is (4 + 5 + 6)/30 = 0.2.

In contrast, using the request filtration strategy, during the above worst case, all the Requests for same content are filtered at the BSs (e.g., requests for the *a* from all MDs are filtered at the *eNB*_2_ PRT). Moreover, the SAE-GW also filters the request from its BSs as shown in Figure 3 (e.g., requests for the *a* from *eNB*_1_ and *eNB*_2_ are filtered at the SAE-GW PRT). Hence, only one request for each content is sent out, where one Request for *d* is delivered to the origin (i.e., 1/30 load origin). If each request arrives after its previous Reply data returns back (the above best case), the request filtration strategy performs as well as the coordinated strategy since no request filtration happens.

In addition, the traffic delivered is measured by the product of average number of network hops and content's size. Based on the assumption of unit-size contents, the traffic delivered can be directly expressed as the number of network hops traversed when fetching contents. Using the noncoordinated strategy, MDs requesting for *a* can directly be satisfied from their accessed BSs without going through any peer BS, while requests for *b*, *c*, and *d* have to go to the origin which is three hops away via SAE-GW (i.e., assume that the hop count is 2 from SAE-GW to origin). Therefore, the average traffic delivered for noncoordinated strategy is (3 · 6 · 3)/30 = 1.8 per request. Using the coordinated strategy, under best case where the requests for *a*, *b*, and *c* are all served in the RAN, only requests for *d* are sent to origin. Hence, the average traffic delivered is [(2 · 5 + 3 · 1)+(2 · 6 + 3 · 1)+(2 · 7 + 3 · 1)]/30 = 1.5 per request. While under the worst case when all requests arrive concurrently, the average traffic delivered is [(2 · 2 + 3 · 4)+(2 · 2 + 3 · 5)+(2 · 2 + 3 · 6)]/30 = 1.9 per request since the unsatisfied *a*, *b*, and *c* have to go to the origin. In contrast, the request filtration strategy perform best in worst case, since all repeating requests are filtered at BSs and SAE-GW, only generating [(2 · 2 + 3 · 1)+(2 · 2 + 1 · 1)+(2 · 2 + 1 · 1)]/30 = 0.56 per request. When no request filtration happens (the above best case), the request filtration strategy performs as well as the coordinated strategy.

In this example, the request filtration strategy leads to a lower load on original server and a lower traffic delivered than both the noncoordinated and coordinated caching strategies. This is not surprising since most repeating traffics are filtered by request filtration. Moreover, if no request filtration happens (i.e., *λ*_*i*_^*k*^ · *τ*_1_≺1), the request filtration strategy performs as well as the coordinated strategy. Furthermore, normally, the coordinated strategy can achieve better performance than the noncoordinated strategy. However, in the case of the above worst case, the coordinated strategy causes more total traffic than the noncoordinated strategy. The reason is that the cached content cannot serve the requests from the peer BSs due to the upstream bandwidth limitation. This key discovery implies that *sometimes the simple noncoordinated strategy can perform better than the elaborative coordinated strategy, especially in the case of heavy request and limited link capacity.* Hence, it is important to investigate the relationship between storage and link capacity to design a proper coordinated caching strategy so as to improve the network performance.

## 4. Bandwidth Allocation under Request Filtration

We next propose a coordinated edge caching strategy based on the request filtration in a RAN, which investigates the cache storage and link capacity cooperatively.

### 4.1. Request Filtration

As explained in the above section, the request filtration can greatly reduce the network traffic delivered. Here we first describe the specific request filtration operation in a RAN. Note that contents are segmented into smaller pieces in this paper, each of which is treated as an individually named content object, to allow flexible distribution and flow control.

First of all, a PRT which records all unsatisfied requests is maintained at each BS and SAE-GW. The PRT has a similar function to that of the Pending Interest Table (PIT) in CCN [27]. An entry in PRT is set up by the first new coming content request. When a request arrives at a node, a longest-match lookup is done on its content name. A PRT match means the content was solicited by request(s) sent by this node. So, if there is an exact-match PRT entry the requesting node (or arrival face) will be added to the PRT entry's “requesting node” list and the request will be discarded (filter the following same request). When the Reply data packet solicited arrives, a copy of that packet will be multicast to all the nodes that the PRT records. We called this procedure asynchronous multicast. Then PRT entries are erased as soon as they have been used to forward a matching Reply data packet (the Reply consumes the request).

An example illustrating the request filtration is shown in Figure 3. When four MDs send requests of content *a* to *eNB*_2_ concurrently, the *eNB*_2_ sends the request to SAE-GW only once, while adding all MDs to its PRT. Furthermore, the requests for content *a* arriving at SAE-GW (from *eNB*_2_ and *eNB*_3_) are also filtered into one request (see the entry for *a* at PRT of SAE-GW), which is sent to the *eNB*_1_ according to the routing table. Then the returning content *a* will be multicast to *eNB*_2_ and *eNB*_3_ at SAE-GW and then multicast to all MDs at each BS instead.

In addition, note that the above asynchronous multicast can be implemented through intercepting the *HTTP GETs* by the aggregating nodes [39] or some other methods such as the content routing in CCN [27], which is out of the research scope of this paper.

### 4.2. Shared Cache Size and Link Bandwidth

As we know, the unsatisfied requests at the access BS always prefer fetching contents from the other caching nodes in the same RAN over the original server. Based on the above request filtration scheme, all these requests will filter again at the SAE-GW. Hence, there is at most one request being sent out at the SAE-GW, to fetch the cached content simultaneously. Therefore, *one copy of the cached content in the same RAN is enough to serve all requests from the peer BSs with the request filtration scheme*.

However, the content cached at the BS does not mean that it can serve the peer requests. How percentage of the cached content can be shared within the RAN is determined by the upstream link capacity. Here we assume the downstream link will not become the bottleneck, compared to the upstream link, which is practical in the real mobile network. Hence, *the shared cache size is tied up with the upstream link bandwidth*.

**Figure 4:**
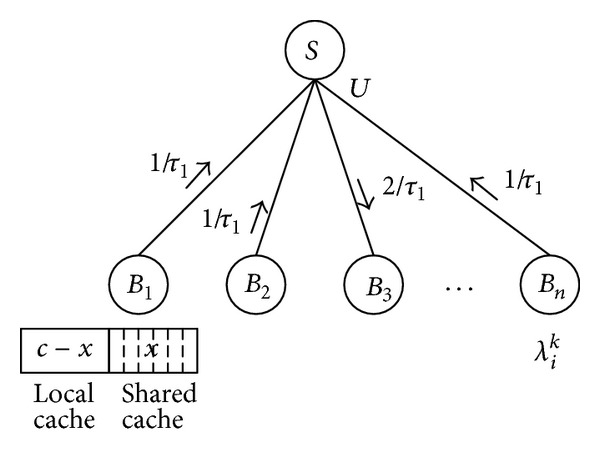
Indirect coordinated edge caching model.

Therefore, we divide the cache storage at the BS into two parts, the noncoordinated *local cache c* − *x* and the coordinated *shared cache x*, as shown in Figure 4. Here we derive the relationship between the shared cache size *x* and its upstream link bandwidth *U*. As explained in Section 3.2, the request filtration happens when requests for the same content arrive at an aggregating node before the previous Reply data for the same content returns back (e.g., *λ*_*i*_^*k*^ · *τ*_1_ ≥ 1 at BS). In this case, only one request is sent out during the latency of fetching the content, so the Request rate sent out from the aggregating BS is reduced to 1/*τ*_1_ no matter how fast the *λ*_*i*_^*k*^ is. Similarly, when all these requests filter again at the SAE-GW, the largest request rate for one content stressed on the link is only 2/*τ*_1_ no matter how many child nodes request this content (we assume the round latency between SAE-GW and BS is *τ*_1_/2). Therefore, based on the assumption of unit-size contents, the maximum required upstream link bandwidth is expressed as

(1) $$\begin{array}{c} U_{\max}=x\cdot \frac{2}{\tau_{1}}. \end{array}$$

Equation (1) gives the relationship between the shared cache size *x* and its upstream link bandwidth *U*; namely, the *U*_max⁡_ *can satisfy all requests for the contents in the shared cache in a RAN, without caring about the request density,* with request filtration.

## 5. Cache Content Placement and Request Routing

In this section, we describe the details of the proposed indirect coordinated edge caching strategy, where the MD's storage capacity is additionally considered as an improvement to the RAN caching system.

### 5.1. Caches on the Users

Since smartphones have large hard disks built in, these devices can effectively act as caches to serve the other users in the same cell. Of course, users need to be incentivized to help other users: in other words, there has to be a compelling answer to the question: why should I spend my battery to provide you with faster video download? Since network operators benefit from offloading of traffic to end users, such incentives can be provided by network operators. They could take on, for example, the form of discounts or increased data caps for participating users, which is out of the scope of this paper.

We assume in the following that the content caching and delivery on the users are controlled by the BS, so that there is a central control unit that has knowledge about which station has what files in its cache and also knows the channel state information (CSI) between the users. This allows a more efficient scheduling of the communications and ensures that there is no interference between the two types of traffic; this is essential for network operators. Therefore, the content, if not cached at the storage in the BS, can be cached in the required user controlled by its access BS. In this way, the total caches in a cell can maximize the supported requests and reduce the backhaul traffic without contents redundancy. The specific caching strategy is explained in the following paragraph.

### 5.2. Content Placement and Request Routing

Based on the above analysis, we allocate the bandwidth in our proposed coordinated edge caching strategy according to (1). To simplify the analysis and without loss of generality, we assume that there are *n* BSs which has the equal cache size *c* and the equal upstream link capacity. Each BS stores in its local cache (i.e., the *c* − *x* portion) the top ranked contents in a noncoordinated manner, and all BSs collaboratively store *n* · *x* contents that are ranked from *c* − *x* + 1 to *c* − *x* + *nx*. In order to manage the coordinated caching across the RAN, the SAE-GW has to collect the information of content popularity and disseminate contents to the corresponding BSs periodically. Hence, the SAE-GW keeps the catalog recording the cached content location.

**Figure 5:**
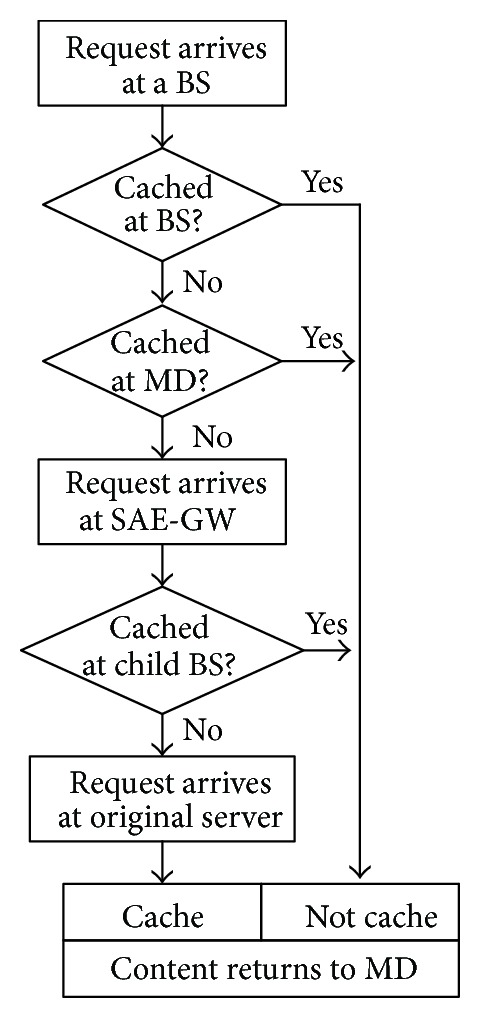
The operation procedure of coordinated edge caching strategy.

The entire operation procedure of coordinated edge caching strategy is then summarized in Figure 5, where the requests are handled following request filtration rule at each node without specific explanation.

Upon receiving a request, the access BS first checks whether it has a copy in its cache or not. The cached copy can serve the MD directly; otherwise, the access BS will check whether its other MDs have a copy in their own caches or not. If another MD has cached the request content and is willing to serve others, the BS will arrange a communication between these two MDs. In the case that there is no copy of the request content cached in the total cell, the request will be sent up to the SAE-GW. Upon receiving a request, the SAE-GW checks whether any child BSs have a copy in their cache or not. If find, fetch the content from its child BS to serve the MD. If not find, fetch the content from the original server.

Upon receiving a Reply, the SAE-GW multicasts it to its child BSs and then the BS multicasts it to its MDs according to PRT. Additionally, if an MD agrees to share its storage with the others, when receiving a content, the BS can inform the required MD to cache it in case of the Reply content coming from the original server. So the BS keeps the MD catalog recording the cached content location. Note that the content caching at the MD's storage is pulled by the user's request, which is different from the content caching at the BS's storage (where content is pushed by the network periodically). Moreover, if the MD moves out of the cell control of the BS, the BS should delete the corresponding content items in its MD catalog through some detections or MD's notification.

With this coordinated edge caching, the next work is to find the optimal *x* in order to improve the network performance, which is addressed in the following section.

## 6. Problem Formulation and Analysis

In this section, we formulate the problem of edge cache division as an optimization problem and study the optimal solution.

### 6.1. Problem Formulation

We consider a simple RAN model, where a set of BSs with storage capability serve content requests originated from its cell, as shown in Figure 3. The original server *O* stores all content objects, referred to as the “origin”; therefore, requests for any content object can always be satisfied by *O*. The other detail model notation is given in Table 2. To simplify the analysis, we also normalize the content size to one unit with respect to nodes' storage capacity. Our objective is to explore the optimal shared cache size *x* for the proposed coordinated edge caching strategy, in order to reduce the average latency user perceived.

Many studies have shown that the content popularity follows the Zipf distribution [40–42]. The Zipf's law predicts that out of a population of *N* elements, the frequency of elements of rank *i*, denoted by *f*(*i*, *α*, *N*), is

(2) $$\begin{array}{c} f\left(i,\alpha ,N\right)=\frac{1/i^{\alpha }}{\sum_{j=1}^{N}\left(1/j^{\alpha }\;\right)}=\frac{1/i^{\alpha }}{H_{N}^{\alpha }},\;i=1,2,.\ldots \end{array}$$

Moreover, we compute the overall probability of requesting for the top *k* contents by

(3) $$\begin{array}{c} F\left(k,\alpha ,N\right)=\sum_{i=1}^{k}f\left(i,\alpha ,N\right)=\frac{H_{k}^{\alpha }}{H_{N}^{\alpha }},\;k=1,2,\ldots , \end{array}$$

where *H*_*k*_^*α*^ and *H*_*N*_^*α*^ are the *k*th and *N*th harmonic numbers of order *α*. In order to ease the analysis and derive meaningful results, we assume that *N* is sufficiently large and approximate *F*(*i*, *α*, *N*) using a continuous function

(4) $$\begin{array}{c} F\left(k,\alpha ,N\right)\approx \frac{\int_{1}^{k}t^{-\alpha }dt}{\int_{1}^{N}t^{-\alpha }dt}=\frac{k^{1-\alpha }-1}{N^{1-\alpha }-1}. \end{array}$$

Therefore, the average latency of serving a content request is expressed as [43]

(5) L(x,α,τ)=F(c−x,α,N)τ0+[F(c−x+xn,α,N)−F(c−x,α,N)]τ1+[1−F(c−x+xn,α,N)]τ2.

We can derive the average latency by adding (4) into (5)

(6) L(x,α,τ)=τ0·(c−x)1−α−1N1−α−1 +τ1·(c−x+nx)1−α−(c−x)1−αN1−α−1 +τ2·(1−(c−x+nx)1−α−1N1−α−1)=1N1−α−1((τ0−τ1)(c−x)1−α       +(τ1−τ2)(c−x+nx)1−α        +τ2N1−α−τ0).

### 6.2. Optimal Strategy

Li et al. provide a rigorous proof of the existence and uniqueness of the optimal strategy in [43]. We solve the optimal solution by letting the first-order derivative of *L*(*x*, *α*, *τ*) equal to zero; that is, ∂*L*(*x*, *α*, *τ*)/∂*x* = 0. So we have

(7) (τ0−τ1)(1−α)(−1)(c−x)−α  +(τ1−τ2)(1−α)(n−1)(c−x+nx)−α=0.

Then we can get

(8) $$\begin{array}{c} \frac{{\left(c-x+nx\right)}^{\alpha }}{{\left(c-x\right)}^{\alpha }}=\frac{\left(\tau_{2}-\tau_{1}\right)}{\left(\tau_{1}-\tau_{0}\right)}\left(n-1\right). \end{array}$$

Here, we introduce a parameter *γ* = (*τ*_2_ − *τ*_1_)/(*τ*_1_ − *τ*_0_) as the ratio of layer latency. Then we can get the optimal shared cache size as

(9) $$\begin{array}{c} x^{*}=c\cdot {\left(1+\frac{n}{\gamma^{-\alpha }{\left(n-1\right)}^{-\alpha }-1}\right)}^{-1}. \end{array}$$

If we use parameter *η* = *x*/*c* to represent the percentage of the local cache size used for shared cache, the optimal solution is

(10) $$\begin{array}{c} \eta^{*}={\left(1+\frac{n}{\gamma^{-\alpha }{\left(n-1\right)}^{-\alpha }-1}\right)}^{-1}. \end{array}$$

It is important to note that η*is a function of the deliver latency, rather than the content request rate *λ*_*i*_^*k*^. Therefore, this scheme is applicable due to low complexity and cost efficiency.

## 7. Performance Evaluation

We test our scheme in a Java-built simulation environment for RAN, by comparing with noncoordinated caching (*non-co*) and coordinated caching (*co*) illustrated in Section 3. In a 3-level topology similar to that in Figure 3, the root node represents the original server node and there are 10 BSs with edge cache under a SAE-GW control.

The original server holds all the contents, and the total size of these edge caches ranged from 15% to 60% of the whole contents. We consider 2000 contents with the same unit size, fetched by 50000 requests, and the content popularity distribution is assumed to be Zipf (*α*) with *α* = 0.9. The bigger *α* indicates that few distinct contents attract the majority of the requests while the smaller ones indicate almost uniform document popularities [40]. Note that we do not consider the cache size provided by the MDs in our simulation, since the users' contribution depends mainly on the number of the MDs and fluctuates according to the users' movement.

Additionally, we give two sets of results derived from different delivery latency *τ*_1_, in order to show the effect of request filtration. However, since our test cannot simulate the real mobile network environment, we only collect data for different filtration interval (i.e., filter per 50 requests and per 300 requests) to analog the different fetching latency for simplicity.

Furthermore, we evaluate how various optimal η* affects the caching performance by changing the *γ* value. We derive the results from two η*values of 1/6 and 5/6, respectively, as the higher the η* is, the larger the shared cache size and the total cached contents at the BSs are.

**Figure 6:**
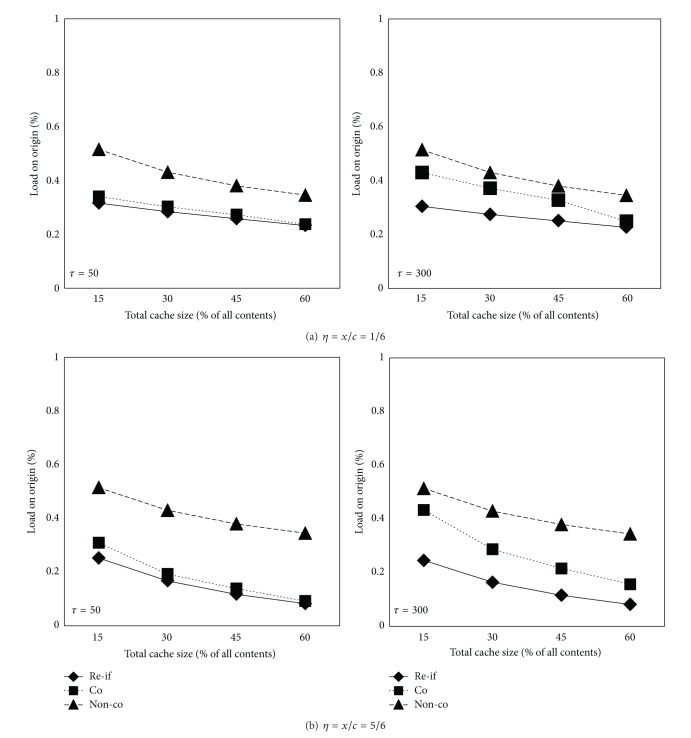
Load on origin versus cache size.

Figure 6 plots the load on original server curves as a function of cache size for different caching schemes. The load on origin is defined as the percentage of all requests served directly by the original server. The proposed request filtration scheme (*re-fi*) improves the load on origin over the other schemes. For example, at cache size 15% (*τ* = 50, η* = 5/6), the load on origin is 25%, 31%, and 51% for *re-fi*, *co*, and *non-co* respectively. In addition, it can be clearly seen that the proposed scheme under the scenario of *τ* = 300 shows a little better performance than that of *τ* = 50. This is because more requests are filtered during the long content fetching latency, while the coordinated caching scheme sends more requests to the origin due to limited link bandwidth.

Moreover, we observe that the load on the origin decreases as η* increases, because the larger total shared cache size is, the more requests are satisfied at the edge BS in a RAN.

**Figure 7:**
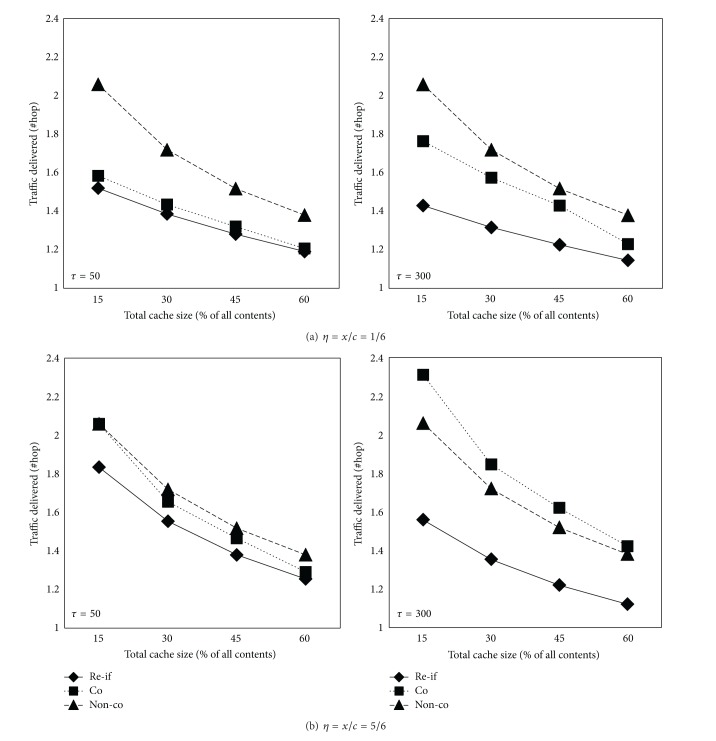
Traffic delivered versus cache size.

Figure 7 shows the average traffic delivered under each scheme, where the traffic is measured in number of hops travelled when fetching the unit-size content. We assume the hop count is 4 when acquiring content from the original server and 2 from the peer BS. The lower the average traffic delivered, the better the performance. The proposed request filtration scheme reduces the total traffic compared to the other schemes. This is not surprising because the proposed scheme can greatly reduce the traffic delivered by request filtration and asynchronous multicast at all nodes in the RAN. Furthermore, we observe the traffic delivered decreases a little as η* increases at *τ* = 300, because the larger total shared cache size is, the more requests are satisfied at the edge BS in a RAN.

Note that under the scenario of *τ* = 300, η* = 5/6, the noncoordinated caching performs better than coordinated caching, as the worst case stated in Section 3.2. This observation reveals that the request rate and the upstream link capacity can seriously impact the desired effect of elaborative coordinated strategy.

**Figure 8:**
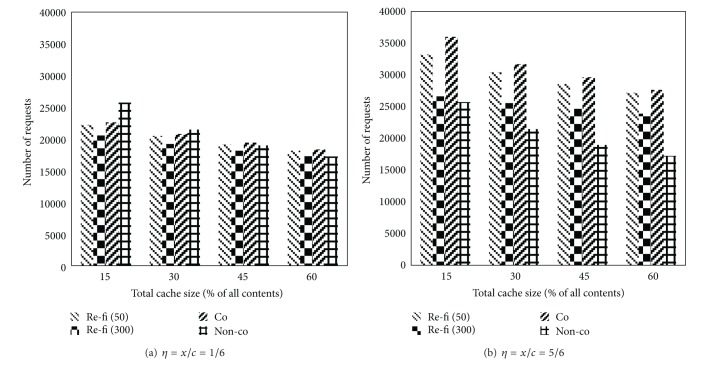
Number of requests versus cache size.

In addition, Figure 8 shows the number of requests sent out by the SAE-GW, either to the original server or to its child BSs. These data reflect how many requests are filtered for the proposed scheme in contrast to the coordinated caching strategy. Hence, fewer requests are sent out in *re-fi* than that in *co*, especially under the scenario of *τ* = 300. Additionally, it is obvious that the number of requests increases as η* decreases, since the larger local cache size can satisfy more requests locally.

However, there are least requests sent out from the SAE-GW in non-co (no request is sent to child BSs in this case), since more requests are satisfied locally due to large local cache size without sharing with others. We also note that the proposed scheme achieves the above merits at a cost of maintaining PRT at all nodes in the RAN.

## 8. Conclusion

Content caching at the BSs is an effective method to improve the mobile network performance. We argued that massive repeating traffics flood the network and impact the coordinated caching effect in this paper. The proposed coordinated caching scheme could greatly reduce the traffic delivered in the network by request filtration and asynchronous multicast. The optimal edge cache size division is also derived in order to reduce the average latency user perceived. The experimental results showed that the proposed scheme effectively reduces the load on the original server and the total traffic delivered through the CN and RAN compared to the existing caching algorithms.

## Figures and Tables

**Table 1:** Comparing the three edge caching strategies.

	Non-coordinated caching	Coordinated caching	Request filtration
Load on origin	0.6	0.1~0.2	0.033~0.1
Traffic delivered	1.8	1.5~1.9	0.56~1.5

**Table 2:** Model notation.

Symbol	Meaning
*n*	Number of BSs
*N*	Number of contents
*c*	Storage capacity of each BS
*x*	Shared cache size at each BS
*α*	Zipf exponent
*τ*_0_	The average latency of serving requests from access BS
*τ*_1_	The average latency of serving requests from peer BS
*τ*_2_	The average latency of serving requests from original server *O*
